# Near full-length genome analysis of low prevalent human immunodeficiency virus type 1 subclade F1 in São Paulo, Brazil

**DOI:** 10.1186/1743-422X-6-78

**Published:** 2009-06-16

**Authors:** Sabri Saeed Sanabani, Évelyn Regina de Souza Pastena, Walter Kleine Neto, Claudia C Barreto, Kelly T Ferrari, Erika MN Kalmar, Suzete Ferreira, Ester Cerdeira Sabino

**Affiliations:** 1 Fundação Pro-Sangue, Hemocentro, São Paulo, Brazil; 2 Retrovirology Laboratory, Federal University of São Paulo, Brazil; 3 Department of Parasitic and Infectious Disease, Faculty of Medicine, University of São Paulo, São Paulo, Brazil; 4 STD/AIDS Reference and Training Center, São Paulo, Brazil

## Abstract

**Background:**

The genetic diversity of the human immunodeficiency virus type 1 (HIV-1) is critical to lay the groundwork for the design of successful drugs or vaccine. In this study we aimed to characterize and define the molecular prevalence of HIV-1 subclade F1 currently circulating in São Paulo, Brazil.

**Methods:**

A total of 36 samples were selected from 888 adult patients residing in São Paulo who had previously been diagnosed in two independent studies in our laboratory as being infected with subclade F1 based on *pol *subgenomic fragment sequencing. Proviral DNA was amplified from the purified genomic DNA of all 36 blood samples by 5 fragments overlapping PCR followed by direct sequencing. Sequence data were obtained from the 5 fragments of pure subclade F1 and phylogenetic trees were constructed and compared with previously published sequences. Subclades F1 that exhibited mosaic structure with other subtypes were omitted from any further analysis

**Results:**

Our methods of fragment amplification and sequencing confirmed that only 5 sequences inferred from *pol *region as subclade F1 also holds true for the genome as a whole and, thus, estimated the true prevalence at 0.56%. The results also showed a single phylogenetic cluster of the Brazilian subclade F1 along with non-Brazilian South American isolates in both subgenomic and the full-length genomes analysis with an overall intrasubtype nucleotide divergence of 6.9%. The nucleotide differences within the South American and Central African F1 strains, in the *C2-C3 env*, were 8.5% and 12.3%, respectively.

**Conclusion:**

All together, our findings showed a surprisingly low prevalence rate of subclade F1 in Brazil and suggest that these isolates originated in Central Africa and subsequently introduced to South America.

## Background

Genetic variability is a major feature of the HIV-1 and considered the key factor to frustrate efforts to halt the virus epidemic. High mutation and replication rates, genomic recombination, therapy and immune-mediated selective pressures are some of the influential forces in the evolution of HIV [1-6]. Approaching this diversity is critical to lay the groundwork for the design of successful drugs or vaccine [7].

Based on the HIV-1 genetic variations and pattern observed in phylogenetic reconstruction, researchers have classified the virus into groups, subtypes and sub-subtypes [8]. Currently, three groups (M, main; O, outlier; N, neither) have so far been recognized. HIV-1 group M viruses are responsible for more than 99% of viral infection worldwide [7] and are further classified into nine (A-D, F-H, J and K) subtypes. Moreover, early sequencing studies have provided evidence of interstrand crossover between genomes of different HIV subtypes [5,6]. Such interclade recombinant strains are consistently reported from regions where two or more clades are predominant. Recombinant strains from unlinked epidemiological sources that exhibit identical patterns of mosaicism have been classified separately as circulating recombinant forms (CRFs) [9]. Up to this writing, there are more than 40 defined CRFs that are epidemiologically important as subtypes . In addition to the known CRFs, a large number of unique recombinant viruses have been characterized worldwide [10]. HIV-1 subtypes, CRFs and URFs show considerably different patterns of distribution in different geographical regions.

On a global scale, the distribution of non-recombinant subtype F1 strains is heterogeneous. For instance, earlier molecular epidemiological studies have detected this subclade in 3–10% of the population in Central Africa [11,12] which is considered the epicenter of the HIV pandemic. Authors of a previous study on a genetic survey of HIV strains from serum samples collected in the mid-1980s from the Democratic republic of Congo (DRC) demonstrated a continuum and remarkably high diversity within and between the F1 and F2 sub-subtypes [13]. In Europe, the genuine subtype F1 strains are by far the most frequent subtype in Romania, representing >70% of the circulating strains among adults and children in this country [14-17]. In addition, a recent study [18] found a close phylogenetic relationship between Angolan and Romanian HIV-1 subtype F1 isolates and thus lent further support to available published data that indicated an African origin of subtype F epidemic in Romania [16,19]. A significantly high proportion of HIV-1 F1 strains were isolated from 11 out of 18 patients infected with non-B viruses of Luxembourg origin [20]. This result led the authors to suggest a small-scale epidemic of F1 viruses among Luxembourg population. In other European countries such as France, Russia and Belgium only sporadic cases of F1 viruses have been documented [21-23]. Predominance of subclade F1 has also been reported in various countries in South America [24-28].

Brazil is the Latin American country that has been badly affected by the HIV epidemic and has the second highest number of HIV-1 cases in the Americas after the USA with an estimated number of 730.000 cases living with HIV at the beginning of 2008 (2008 Report on the Global AIDS Epidemic). HIV-1 subtype B is a major genetic clade circulating in the country. However, existences of small proportion of other subtypes such as F1, C, B/C and B/F have been consistently reported [28-30]. Data from recent studies of full genomic characterization of HIV have provided evidence of Brazilian CRF strains designated as CRF28_BF, CRF29_BF, CRF39_BF, CRF40_BF and CRF31_BC [30-33].

Sub-subtype F1 is considered the main non-B subtype circulating in the country. However, our recent data indicated low proportion of HIV-1 F1 in Brazil than previously thought and assumed the replacement of genuine sub-subtype F1 by emerging BF1 recombinants as a result of unknown selective advantage [30]. Most of the published sequences of HIV-1 F1 strains in Brazil were based on small genetic stretches and only five near full-length genomes (NFLG) have so far been characterized. In this study, we aimed to provide new genetic materials of this subclade by sequencing their NFLG and attempt to define its prevalence in the state of São Paulo, the most populous city located in the southeast region of Brazil.

## Methods

### Study population

A total of 36 samples were selected from adult patients residing in São Paulo who had previously been diagnosed in two independent unpublished studies in our laboratory as being infected with HIV-1 F1 based on *pol *subgenomic fragment sequencing. The first study investigated the long-term therapeutic interruption and genetic survey of HIV-1 variants in 137 patient samples collected in 2002 and identified 8 HIV-1 F1 (5.8%) in their group. The second study assessed the genotypic resistance and virus distributions in a cohort of 751 HIV-1 infected patients on antiretroviral therapy between 2006–2007 and found a total of 28 patients (3.7%) infected with sub-subtype F1. All of the 36 samples initially characterized by both projects were submitted for NFLG.

### Amplification and sequencing of HIV-1 DNA

Genomic DNA was extracted from peripheral blood mononuclear cells (PBMCs) using the QIAamp blood kit (Qiagen GmbH, Hilden, Germany) according to the manufacturer's instructions. Extracted gnomic DNA concentration of the samples under investigation were determined by comparing the samples band relative to the band of the comparable intensity in the high DNA mass ladder (Invitrogen, Brazil) using 0.5–1.0% agarose gel. All extracted genomic DNA were submitted for amplification of a house keeping gene (β-globin) with primers PC03 and PCO4 [34] to examine DNA integrity and exclude the presence of PCR inhibitors. Proviral DNA was amplified from the purified genomic DNA by PCR using primers and methods described in previous studies [29,30]. Amplification reactions were done in duplicate to eliminate PCR artifacts, ensuring that sequenced NFLG were not assembled from heterogeneous DNA targets. Both DNA complementary strands were sequenced directly from purified PCR products by using variety of internal primers, fluorescent-dye terminators, and *Taq *polymerase on an automated sequencer (ABI 3130, Applied Biosystems Inc., Foster City, CA).

### Subtype classification and sequence analysis

The data from each sequenced fragment were edited and initially screened for the presence of recombination patterns by the REGA HIV-1 subtyping tool (v2) [35] and the jumping profile Hidden Markov Model (jpHMM) [36] and further confirmed by using the bootscanning method [37] implemented in SimPlot 3.5.1 for Windows [38] using the following parameters, window size 250 bp, step size 20 bp and the Kimura 2-parameter as a model to estimate nucleotide substitution. The significant threshold for the bootscan was set at 90%. This strategy has allowed us to early identify samples with any recombinant fragments and to exclude them from further experiments and analyses. Only edited subclade F1 fragments were assembled into contiguous sequences on a minimum overlap of 30 bp with a 99–100% minimal mismatch and a consensus sequence was formed by the Sequencher program (Gene Code Corp., Ann Arbor, MI). NFLG consensus sequences were again analyzed for recombination by using the jpHMM web tool. Only jpHMM confirmed pure sub- subtype F1 isolates were aligned with reference sequences representing subtypes A-D, F-H, J and K obtained from the Los Alamos database  using the CLUSTAL X program [39]. Aligned sequences were further refined by manual editing and trimmed to the minimal shared length in the BioEdit Sequence Alignment Editor Program [40]. Gaps and ambiguous positions were removed from alignment. The phylogenetic trees were constructed by the maximum likelihood (ML) methods implemented in the program PHYML [41] using the GTR + I + G substitution model and a BIONJ starting tree. Heuristic tree searches under the ML optimality criterion were performed using the NNI branch-swapping algorithm. The approximate likelihood-ratio test (aLRT) based on a Shimodaira-Hasegawa-like procedure was used as a statistical test to calculate branch support. Only aLRT statistical values of >70% were considered significant and displayed at the tree nodes. Trees were plotted using the program MEGA version 4 . The mean genetic nucleotide distances within and between sequences were calculated using the maximum composite likelihood model implemented in MEGA version 4.0 software. Comparison of tree topology from subclade F1 subgenomic regions was performed by the recently described algorithm [42]. Prediction of HIV-1 coreceptor usage from the *env *V3 region was determined using the web-based service geno2pheno [coreceptor]  which considers all V3 mutational patterns, and not only changes of arginine or lysine at positions 11 or 25.

### GenBank accession numbers

GenBank accession numbers for the proviral NFLG sequences reported in this study are [02BR082: FJ771006, 02BR170: FJ771007, 06BR564: FJ771008, 06BR579: FJ771009, 07BR844: FJ771010].

## Results

### Phylogenetic analysis of partial pol sequences

**Figure 1:**
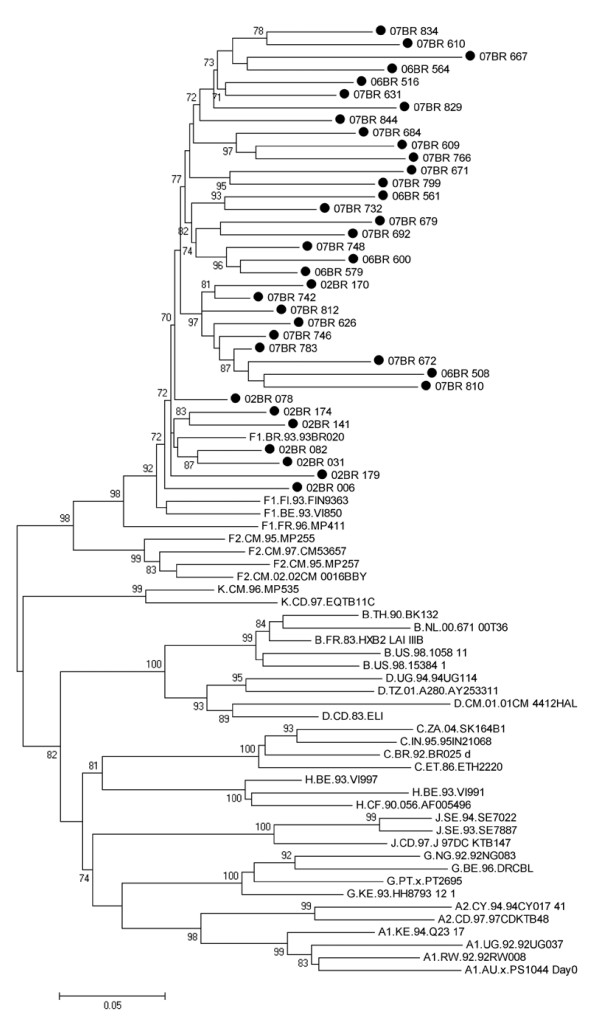
**Maximum likelihood tree of Brazilian subclade F1 strains and reference strains inferred from partial *pol *region**. Black circles show the newly sampled strains from Brazil. For clarity purposes, the tree was midpoint rooted. The approximate likelihood-ratio test (aLRT) values of ≥ 70% are indicated at nodes. The scale indicates the number of substitutions per site.

Of the 888 subjects in total, 36 were initially assigned as infected with non-recombinant HIV-1 subclade F1 variants using approximately 1200 bp region in the *pol *gene as depicted in figure 1. Thus, based on the analysis of this small portion of viral genome, subclade F1 is still present in São Paulo and roughly accounts for 4.05% of circulating strains.

### Analysis of full-length subtype F1 sequences

**Figure 2:**
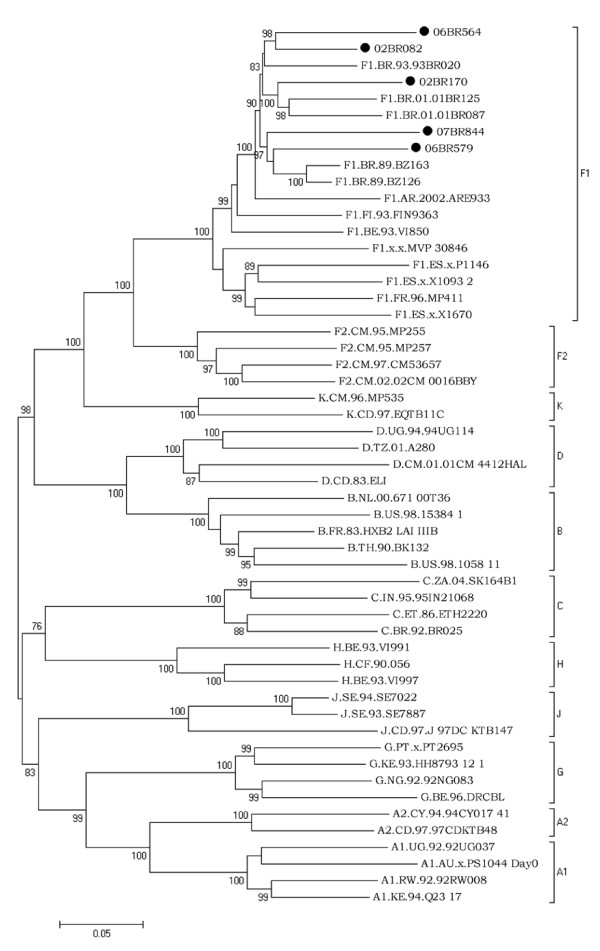
**Maximum likelihood tree of Brazilian subclade F1 strains and reference strains inferred from nearly full-length HIV-1 sequences**. Black circles show the newly sampled strains from Brazil. Nucleotide sequences were compared with reference sequences of subtype A-D, F-H, J and K . For clarity purposes, the tree was midpoint rooted. The approximate likelihood-ratio test (aLRT) values of ≥ 70% are indicated at nodes. The scale bar represents 0.05 nucleotide substitution per site.

**Figure 3:**
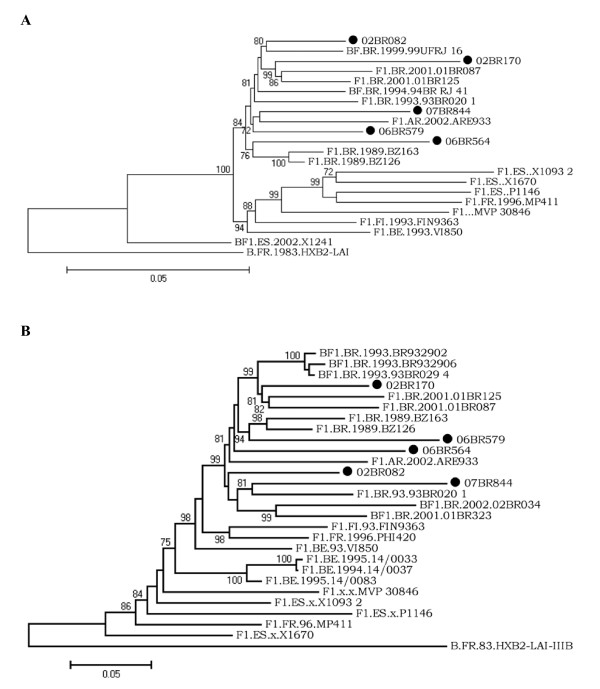
**Maximum likelihood tree of Brazilian subclade F1 strains and reference strains inferred from full-length *gag-pol *(A) and *env *(B) reading frames**. Black circles show the newly sampled strains from Brazil. Trees were rooted using HIV-1 HXB2 isolate. The approximate likelihood-ratio test (aLRT) values of ≥ 70% are indicated at nodes. The scale bar represents 0.05 nucleotide substitution per site.

To find the true prevalence of this strain, all the 36 samples were subjected to complete genome amplification and only genuine HIV-1 F1 strains were corroborated by further phylogenetic analysis of the complete coding sequences and part of LTR region. Of the 36 patients investigated, chimeric viruses comprised of B/F1 and F1/C based on fragment analysis were detected in 31 patients and were omitted from further analysis. Only 5 sequences inferred from *pol *region as subclade F1 also holds true for the genome as a whole and, thus, estimated the true prevalence of HIV FI at 0.56%. Inspection of coding regions of the five HIV-1 F1 sequences obtained in this study displayed open and intact reading frames for majority of HIV proteins. To exclude laboratory strains contamination, a BLAST search of GenBank HIV-1 sequences did not reveal any evidence for contamination with strains obtained from our patients. Figure 2 shows the ML tree of 53 complete genome coding sequences, including the five isolates sequenced in this study and 48 reference strains (GenBank and Los Alamos database) representing subtypes A-D, F-H, J and K. The five isolates, namely 02BR082, 02BR170, 06BR564, 06BR579 and 07BR844, fell into subclade F1 reference group (100% aLRT), with an overall average distance of 6.9%. Close inspection of figure 2 shows that strains clustered in subclade F1 are divided further into two major groups, in which the Brazilian F1 sequences, FI.93.FIN9363 and BE.93.VI850 fell into the first group and F1.FR.96.MP411, F1.ES.x.P1146, F1.ES.x.X1093 2, F1.ES.x.X1670 and F1.x.x.MVP 30846 fell into the other. Within the first group that includes the Brazilian F1 sequences, FI.93.FIN9363, BE.93.VI850 and the Argentinean F1.AR.2002.ARE933 isolate, where the node within this cluster had 99% of aLRT statistical support, there is an indication of two major clusters of the Brazilian sequences separated by 90% of aLRT values. To test the stability and branching orders of our sequences, ML trees were independently made from all the full length HIV-1 F1 *gag-pol *(n = 21) and *env *sequences (n = 27) available in the database (Figure 3a &3b). The phylogenetic trees from both regions received an overall topological score of 69.3%. Consistent with the results obtained by NFLG, all the Brazilian isolates along with other South American (SAm) sequences formed a distinct cluster supported by aLRT value of greater than 80% in both regions as depicted in figure 3. Furthermore, close looking at the SAm sequences in figure 3a and 3b revealed two main clusters separated from each other by 84% and 99% of aLRT values in the *gag-pol *and *env *regions, respectively, and indicated possible intrasubtype recombination. For example, isolate 06BR579 placed the *gag-pol *region within the 07BR844 and F1.AR.2002.ARE933 cluster in 72% of aLRT values, but the *env *region grouped within other cluster that included F1.BR.1989.BZ163 and F1.BR.1989.BZ126 (aLRT 94%). The computed topological score of this cluster in both regions was 50% with branch length mismatch of 59.7%. Therefore, the shifting of topological positions into 2 different phylogenetic trees is suggestive evidence of intrasubtype recombination event or other factors such as convergence.

**Figure 4:**
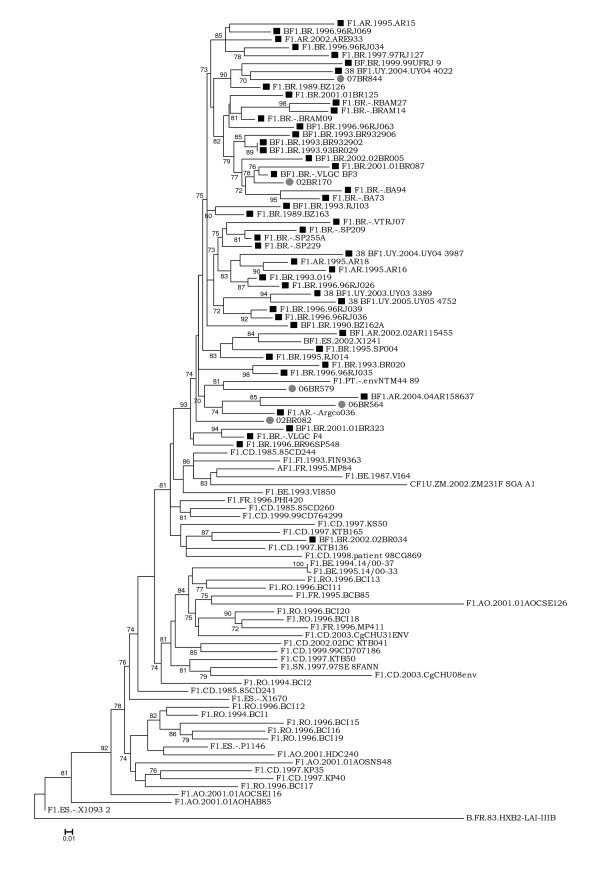
**Maximum likelihood tree of South American F1 and BF1 recombinant strains (Black squares) and reference strains (location is shown in each sequence name) inferred from *C2-C3 env *region**. Gray circles show the newly sampled strains from Brazil. Trees were rooted using HIV-1 HXB2 isolate. The approximate likelihood-ratio test (aLRT) values of ≥ 70% are indicated at nodes. The scale bar represents 0.01 nucleotide substitution per site.

To establish the relationship of our sequences to each other and to the previously published HIV-1 F1 sequences from a variety of geographic regions, another phylogenetic tree was constructed using the *C2-C3 env*. This region was selected because it contains a larger number of HIV-1 F1 sequences in the database. As shown in figure 4, except for isolate 02BR034 (Accession DQ358812), the sequences from SAm including the new isolates in this study emerge from a common node along with two strains, one isolated in Spain from an Argentinean immigrant (BF1.ES.2002.X1241) and the other from Portuguese women (F1.PT.-.envNTM44 89) with unknown epidemiological link to any SAm country. The Brazilian F1 isolates did not form a rigid cluster but were dispersed among the SAm non-Brazilian sequences and were distinct from the Romanian and African isolates. In contrast, the Brazilian strain 02BR034, which branches well out of the SAm cluster, is positioned in the phylogenetic tree close to other isolates from the DRC. Moreover, the branching patterns indicate that the common ancestor of the SAm cluster was also shared with isolates belonging to DRC.

The mean genetic nucleotide distances of the SAm HIV-1 F1 sequences derived from the C2-C3 region were computed and compared with reference strains from Romania and Central Africa (Angola and DRC). The results showed that the SAm isolates differed from the Romanian and Central African F1 strains by nucleotide distances of 12.8% and 12.3%, respectively. The mean nucleotide variation of 8.5%, 11.2% and 12.3% was observed among F1 strains from SAm, Romania and Central Africa, respectively.

### Amino acids and LTR nucleotides alignment features

Detailed inspection of the amino acid alignment of the obtained isolates and the Brazilian subtype F1 reference genomes showed that strains 07BR844 and 01BR087 (Accession DQ358801) had 18 bp insertional mutation in the N-terminus of the p6^Gag ^protein that created a duplication of PTAPP motifs. The same sequences showed concomitant insertion of 6–7 amino acids within the p^6Pol ^epitope (NSPTRREL) with particularly conserved repeat of SPT amino acids. Analysis of the phenotypic characteristics of the 3^rd ^(V3) region in the *env *gene of the new Brazilian subtype F1 isolates described in this study suggests that all, except the isolate 07BR844, were derived from macrophage tropic R5 viruses. The GPG motif at the tip of the V3 loop and the potential N-linked glycosylation sites were highly conserved.

**Figure 5:**
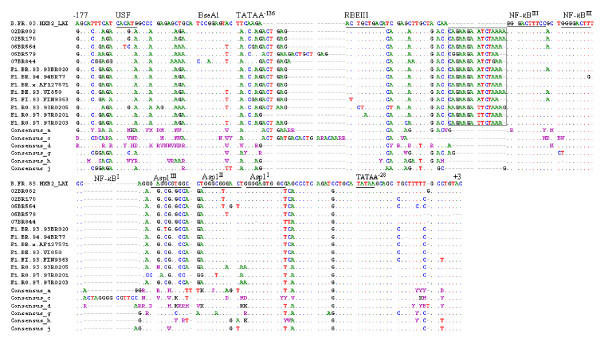
**Alignment of the nucleotide sequences within the LTR region spanning positions -177 to +3 of subclade F1 strains with those of the HXB2_LAI_IIIB_BRU (K03455) HXB2 and consensus sequences of clades a, c, d, g, h and J**. Dots indicate nucleotide identity to the HXB2 sequence and dashes (-) represent gaps introduced to achieve the best alignment. Motifs present in the HXB2_LAI_IIIB_BRU (K03455) are underlined. Boxed sequences in subclade F1 isolates indicated 13–15 nucleotides insertion.

A detailed scrutinization of the partial nucleotide alignment of the 3' LTR regions relative to HXB2 and consensus sequences of other HIV subtypes is shown in figure 5. Conform to the consensus sequence GGGRNNYYCC, two potential NF-κB binding sites were localized in our five new strains. A subclade F1 specific insert of 13–15 nucleotides was observed just downstream of the NF-κB^III ^binding site. The existence of this insertional nucleotides signature has also been reported in a previous study [43]

## Discussion

Currently there are only 13 NFLG sequences of pure subclade F1 isolates in GenBank. Among these were five recovered from Brazil. The scarcity of these sequences prompted us to characterize and provide newer genetic materials of this subclade, which is become rarely found in the Brazilian epidemic. Using our strategy detailed in the material and methods, we attempted to roughly define the molecular prevalence of the non-recombinant subclade F1 in São Paulo, Brazil. Our results confirmed our previous findings [30] and showed a surprisingly low prevalence rate of 0.56% suggesting that, in previous studies, occurrence has been overestimated due to partial genome sequence data [28,44]. Results from a recent study conducted by [45] have suggested that subclade F1 was readily and completely assimilated into the previous (caused by subtype B) HIV-1 epidemics in Brazil. In contrast to this study, our results provided compelling evidence of circulation of subclade F1 in Brazil and, consistent with our previous findings, suggest that recombination of HIV-1 F1 with other circulating clades, particularly subtype B, is on the rise and may, possibly, gradually replace sub-subtype F1 in the future.

The epidemic history of subclade F1 in Brazil with methods based on coalescent theory, using partial and complete sequences of contemporary viruses, indicated that the epidemic growth of this subclades started in the late 1970s and experienced a rapid expansion over the first 10 years, then slows considerably around 1980s [46,47]. It has been suggested that transmission network such as homo/bisexuals and injecting drug users could have contributed to its spread in the 1980–1993 [46,47]. The extremely low prevalence of subclade F1 reported here lends further support to the slow-down of the growth rate estimated in the later study. The subsequent decline of growth rate of this subclade was associated with increased isolation of BF1 recombinant variants in the Brazilian epidemics. We believed that the main evolutionary driving force behind the decline and probably replacement of subclade F1 and emergence of BF recombinants isolate might be due to stochastic events facilitated by the lower population size of the genuine subclade F1 rather than an increased viral fitness or selective pressure.

Several studies showed that subclade F1 from SAm viruses form a monophyletic cluster either by partial or NFLG when compared to other viruses of the same subtype from other countries [30,47]. Our current results were in line with these findings and showed strong cluster of all the SAm in a NFLG tree and this cluster remained unaffected in the *gag-pol *and *env *subgenomic trees. Moreover, pairwise comparison of the SAm cluster in both subgenomic trees provided suggestive evidence of intraclade recombination; however, these findings may need to be explored better with more sequences and robust phylogenetic parametric bootstrapping method.

The placement of the Brazilian strain 02BR034 outside the SAm cluster as shown in figure 4 is striking. This isolate is a B/F1 mosaic virus that is closely related to 01BR323 all across the genome and it has a near identical mosaic structure to 01BR323, except for a short stretch between positions 7000 and 8000 where 02BR034 clusters with subtype B rather than with subclade F1 as previously reported [30]. However, when more subclade F1 *C2–C3 *sequences was then included as shown in figure 4, 02BR034 did not come out as most similar to 01BR323 in this region. One explanation for the aberrant clustering of 02BR034 is either an accidental similarity to subclade F1 from DRC or an additional intrasubtype recombination event involving small region in the *C2-C3*. The overall result presented in figure 4 suggests that the HIV-1 F1 viruses had probably been brought to SAm from Central Africa by a single or a very small group of individuals infected with genetically related viruses then spread from person to person till it took firm hold in the current epidemic. Our findings in this respect are similar to those of Aulicino et al., (2007) in that the founder virus of this subclade has been introduced to SAm from Central African countries.

The duplication of the PTAPP motifs in the p6^Gag ^together with amino acids insertion within the p^6Pol ^epitope were observed in isolates 07BR844 and 01BR087 (previously characterized by our group). The PTAPP is a known binding site of the cellular protein Tsg101 which is involved in intracellular trafficking of plasma membrane-associated proteins [48,49]. It has been shown that duplication of the PTAPP late protein can considerably affect the Tsg101 binding and consequently, alter the rate of HIV release from the membrane [49]. Moreover, a recent study conducted by Cao and co-workers [50] have shown that the duplication of the PTAPP motifs and the mutagenesis of the *pol *NL8 epitope represent novel mechanism whereby HIV-1 can alter its sequence with potential functional consequences for viral replication and budding.

## Conclusion

Finally our study provided concrete evidence of the existence of pure subclade F1 in Brazil and suggests that monitoring of the diversity of the HIV-1 subtypes is extremely relevant to guide diagnosis, treatment and vaccine development. Moreover, we believe that the reported data will be useful as a reference for future studies on the genetic diversity of HIV-1.

## Competing interests

The authors declare that they have no competing interests.

## Authors' contributions

SS conceived and designed the study, did the data analysis of the sequences, and wrote the first draft of the manuscript. ÉRP conducted the characterization of the full-length genome analysis. WKN help with the laboratory work. CCB, KTF, EMNK and SF performed the initial characterization of samples in the *pol *region. ECS designed and directed the study
